# High-resolution genomic profiling of human papillomavirus-associated vulval neoplasia

**DOI:** 10.1038/sj.bjc.6605589

**Published:** 2010-03-16

**Authors:** K J Purdie, C A Harwood, K Gibbon, T Chaplin, B D Young, J B Cazier, N Singh, I M Leigh, C M Proby

**Affiliations:** 1 Centre for Cutaneous Research, Institute of Cell and Molecular Science, Barts and the London School of Medicine and Dentistry, Queen Mary University of London, 4 Newark Street, London E1 2AT, UK; 2 Department of Dermatology, Barts and the London National Health Service Trust, 2nd Floor Outpatients Building, Stepney Way, Whitechapel, London E1 1BB, UK; 3 Cancer Research UK Medical Oncology Laboratory, Institute of Cancer, Barts and the London School of Medicine and Dentistry, Queen Mary University of London, Charterhouse Square, London EC1M 6BQ, UK; 4 Wellcome Trust Centre for Human Genetics, University of Oxford, Roosevelt Drive, Oxford OX3 7BN, UK; 5 Department of Cellular Pathology, Barts and the London National Health Service Trust, Pathology and Pharmacy Building, 80 Newark Street, London E1 2ES, UK; 6 Division of Medical Sciences, College of Medicine, Dentistry and Nursing, University of Dundee, Ninewells Hospital, Dundee DD1 9SY, UK

**Keywords:** SNP array, vulva, VIN, VSCC, human papillomavirus, chromosome aberration

## Abstract

**Background:**

The incidence of human papillomavirus-associated vulval neoplasia is increasing worldwide; yet the associated genetic changes remain poorly understood.

**Methods:**

We have used single-nucleotide polymorphism microarray analysis to perform the first high-resolution investigation of genome-wide allelic imbalance in vulval neoplasia. Our sample series comprised 21 high-grade vulval intraepithelial neoplasia and 6 vulval squamous cell carcinomas, with paired non-lesional samples used to adjust for normal copy number variation.

**Results:**

Overall the most common recurrent aberrations were gains at 1p and 20, with the most frequent deletions observed at 2q, 3p and 10. Copy-neutral loss of heterozygosity at 6p was a recurrent event in vulval intraepithelial neoplasia. The pattern of genetic alterations differed from the characteristic changes we previously identified in cutaneous squamous cell carcinomas. Vulval neoplasia samples did not exhibit gain at 5p, a frequent recurrent aberration in a series of cervical tumours analysed elsewhere using an identical protocol.

**Conclusion:**

This series of 27 vulval samples comprises the largest systematic genome-wide analysis of vulval neoplasia performed to date. Despite shared papillomavirus status and regional proximity, our data suggest that the frequency of certain genetic alterations may differ in vulval and cervical tumours.

Vulval squamous cell carcinoma (VSCC) is a relatively uncommon malignancy in women, accounting for less than 5% of gynaecological cancer (Landis *et al*, 1999). It is now established that there are two distinct types of VSCCs, those associated with high-risk *α* human papillomavirus (hrHPV) and non-hrHPV-associated variants (McCluggage, 2009). HPV-positive tumours are frequently contiguous with classic vulval intraepithelial neoplasia (VIN) and usually arise in younger women. The incidence of HPV-positive VIN and VSCC is steadily increasing worldwide (Joura *et al*, 2000). An association has been reported (Hording *et al*, 1996; de Bie *et al*, 2009) between the development of classic VIN/VSCC and cervical intraepithelial neoplasia (CIN)/cervical SCC (CxSCC) but little is known of whether, in addition to their positive HPV status and regional proximity, these lesions share genetic similarities.

Limited data are available on the genetic alterations associated with VSCC and VIN precursor lesions; previous studies have used microsatellite marker analysis and metaphase comparative genomic hybridisation (CGH) rather than higher resolution array-based techniques (Pinto *et al*, 1999; Jee *et al*, 2001; Rosenthal *et al*, 2001; Allen *et al*, 2002; Micci *et al*, 2003; Bryndorf *et al*, 2004; Huang *et al*, 2005). In contrast, the genetic changes associated with CIN and CxSCC have been extensively studied, most recently using array-based methods (Hidalgo *et al*, 2005; Wilting *et al*, 2006, 2009; Choi *et al*, 2007; Kloth *et al*, 2007; Scotto *et al*, 2008a, 2008b).

Microarray-based single-nucleotide polymorphism (SNP) analysis facilitates the accurate and rapid identification of genome-wide allelic changes in tumour DNA samples. It has been used to investigate the molecular pathogenesis of various human cancers, including cutaneous SCC (Purdie *et al*, 2007, 2009) and cervical cancer (Kloth *et al*, 2007; Scotto *et al*, 2008a, 2008b). Here, for the first time, we have applied high-resolution microarray analysis to vulval squamous neoplasia to compare the pattern of genetic changes occurring during the development of hrHPV-associated VSCC with those alterations previously identified during cervical carcinogenesis. This reported investigation of 27 VIN and VSCC samples is the largest systematic genome-wide analysis of vulval cancer performed to date.

## Materials and methods

### Study patients

Patients attending a dedicated vulval clinic provided written, informed consent to undergo a diagnostic or therapeutic biopsy for either VIN or VSCC. Additional written consent was obtained for two 4-mm punch biopsies to be taken, one from the lesion and one from upper inner arm skin. Full demographic information was collected through a detailed questionnaire. Ethical approval for this investigation was obtained from the East London and City Health Authority local ethics committee and the study was conducted according to the Declaration of Helsinki Principles. All patients biopsied have remained under consultant dermatologist care (KG). Average duration of follow-up is 5 years (range 2–7 years).

### Samples

Routine histopathological evaluation was performed on formalin-fixed vulval lesional sections stained with hematoxylin and eosin, with only samples identified as classic VIN or classic VIN-associated SCC selected for inclusion in this study. Staging of SCC was performed according to Federation of Gynaecology and Obstetrics and American Joint Committee on Cancer TNM criteria, in which Stage I/II is defined as carcinoma confined to the vulva and perineum, whereas Stage III/IV tumours are those that have spread to another anogenital region and/or regional lymph node(s). All diagnoses were confirmed by a single experienced gynaecological pathologist (NS). All punch biopsies collected were immediately snap-frozen in liquid nitrogen.

### Microdissection and DNA extraction

Lesional tissue was microdissected to overcome the problem of stromal/lymphocyte contamination that can lead to a reduction in tumour DNA purity. Laser capture microdissection was performed using the PALM Microbeam system (Carl Zeiss, Welwyn Garden City, Hertfordshire, UK) on 8 *μ*m fresh snap-frozen tissue sections cut onto Zeiss 1.0 PEN membrane slides (Carl Zeiss). DNA was extracted from lesional and non-lesional control skin biopsies using the Qiagen DNA Micro Kit (Qiagen, Crawley, West Sussex, UK) according to the manufacturer's instructions.

### HPV detection and genotyping

The presence of hrHPV DNA was investigated in vulval samples using the digene Reverse Hybridisation Assay (Qiagen) that detects 18 high-risk mucosotropic HPV types. This study focused on HPV-associated vulval neoplasia, hence only hrHPV-positive samples were selected for inclusion in this study.

### SNP array analysis

Samples were subjected to 250 K Nsp SNP array analysis (Affymetrix, Santa Clara, CA, USA) and the proprietary Genome Oriented Laboratory File (GOLF) system was used for the analysis and display of SNP call and signal data as previously described (Purdie *et al*, 2007).

### Comparison with cervical cancer data

250 K Nsp SNP array data from a previously published study on cervical carcinomas (Scotto *et al*, 2008b) were downloaded from the NCBI GEO database (Edgar *et al*, 2002; GEO accession GSE10092) and analysed by GOLF software. This study was chosen for comparative purposes because the experimental protocol mirrored the current investigation, both in terms of the use of fresh frozen samples and the array-based technique. Furthermore, detailed clinical and histological information, including tumour stage, were available for the samples. Data from paired non-lesional samples were not available for analysis, hence raw data from seven normal cervical epithelial samples analysed by Scotto *et al* (2008b) were combined to make a pooled normal control. Vulval squamous cell carcinoma samples were reanalysed similarly and the results compared with those from paired sample analysis to confirm that this approach permitted the accurate identification of gross chromosomal aberrations.

### Statistical analysis

The two-sided Fisher's exact test was used to calculate *P*-values, which were adjusted for multiple testing using Hochberg's method (Hochberg, 1988).

## Results

### Majority of HPV-positive vulval lesions contained HPV-16

The presence of hrHPV DNA was detected in 27 vulval lesional samples, comprising 21 classic high-grade VIN (VIN2/3) and 6 VIN-associated VSCC (all Stage IA/B). HPV-16 was the most frequent type, identified in 90% (19 out of 21) VIN samples as well as in 33% (2 out of 6) VSCC samples (Supplementary Table 1). HPV-33 was detected in three samples (two VIN, one VSCC) and HPV-18 was identified in one VSCC lesion. Lesions were associated with a single HPV type with the exception of 2 multiply-infected VSCC that contained HPV types 45 and 56 and types 39, 58, 59 and 66, respectively. Two high-grade VIN samples tested negative for HPV DNA and were excluded from further analysis.

### Gain events were the most frequent recurrent chromosomal aberrations

**Figure 1:**
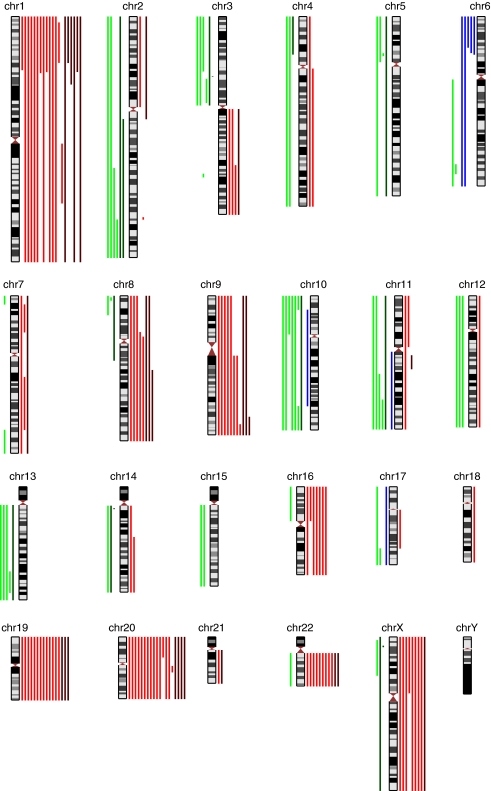
Ideogram summarising allelic imbalance in high-grade VIN (light coloured lines) and VSCC (dark coloured lines). Loss of heterozygosity events are indicated to the left of chromosomes with deletion shown in green and uniparental disomy in blue and gains are indicated to the right in red.

**Figure 2:**
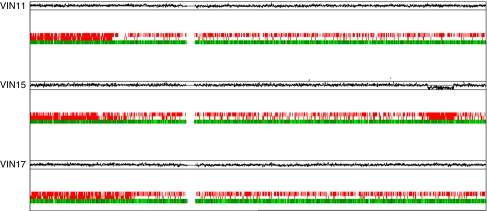
Copy-neutral LOH at chromosome 6pter-6p21.3 in three high-grade VIN samples. Upper panels indicate lesional/non-lesional copy number ratios plotted on a log_2_ scale according to chromosomal position. Upper line represents log_2_(2) and lower line represents log_2_(0.5). Lower panels depict a comparison of SNP genotypes in VIN and paired normal samples. Single-nucleotide polymorphism loci shown in green indicate calls conserved between the two samples, loci shown in red immediately above indicate LOH in the VIN3 sample and loci shown in red on the top row indicate SNPs not called in the VIN3 sample. Localised areas of low call rates may imply LOH in a subpopulation of cells. Note that sample VIN 15 additionally exhibits LOH accompanied by loss of copy number (deletion) at 6q24.3-6q25.3.

SNP microarray analysis was performed on lesional and paired non-lesional DNA samples to adjust for normal copy number variation (Redon *et al*, 2006). In high-grade VIN, gains at chromosomes 1p, 19 and 20 were the most frequent recurrent events, occurring in a majority of samples (13 out of 21, 62% for 1p and 19 and 14 out of 21, 67% for 20; Table 1, Figure 1). Some recurrent losses were observed, with the most frequent occurring at 10p (6 out of 21, 29%), 10q (5 out of 21, 24%) and 2q, 3p and 11q (all 4 out of 21, 19% samples). Comparison of SNP genotypes in lesional and paired normal samples revealed that 5 of 21 samples (24%) displayed extensive loss of heterozygosity (LOH) at 6p in the absence of copy number change. In three cases this copy-neutral LOH, also known as uniparental disomy (UPD), involved the distal region from 6pter-6p21.3 (Figure 2). The remaining two samples showed UPD across the whole of chromosome 6. In VSCC, gains remained the most frequent events, with all six samples showing 1p gain (Table 1, Figure 1). Gain of whole chromosome 20 was observed in four of six samples (67%) and gains at 1q, 8q, 9q and whole chromosome 19 all occurred in three samples (50%). Recurrent losses occurred at 2q, 3p, 14q and Xp, all in two of six (33%) samples.

### Most frequent recurrent aberration in CxSCC was not detected in vulval neoplasia

To examine whether the genetic alterations observed here were shared by other HPV-associated genital neoplasia, we used the same software platform to analyse previously published data from a series of 70 cervical carcinomas that had been subjected to an identical microarray analysis protocol (Scotto *et al*, 2008b). Final analysis was restricted to 53 HPV-positive CxSCC with high call rates, including 10 tumours classified as Stage IA/IB according to Federation of Gynaecology and Obstetrics cervical carcinoma staging criteria (defined as carcinoma confined to the cervix). Gain at 5p, which had been identified as the most frequent recurrent change in the series of 70 CxSCC (Scotto *et al*, 2008b), was observed in 58% (31 out of 53) of the re-analysed cervical tumours compared with none of the 27 vulval neoplasia samples. Statistical analysis revealed that this aberration was significantly more frequent in CxSCC than VSCC, *P*-value=0.0083. The difference between the two groups did not reflect the earlier tumour stage of the VSCC series because this event occurred at comparable frequency in Stage I cervical tumours (50% Table 2). Other differences between the two tumour groups were not significant at the 5% level after adjustment for multiple testing.

## Discussion

This study is the first to investigate the genome-wide genetic changes in HPV-associated VIN and VSCC using high-resolution SNP array analysis. In a series of 27 VIN/VSCC samples with matched normal tissue, we have shown gains to be the most frequent chromosomal aberrations in both VIN and VSCC. The most common recurrent aberrations were gains, at 1p and 20 in particular. Some recurrent deletions were also observed, most frequently at 2q, 3p and 10. Copy-neutral LOH at 6p was a recurrent event in VIN. Despite our use of high-resolution SNP analysis, gross chromosomal aberrations comprised the majority of the recurrent events, with a few exceptions such as 3p and14q microdeletions, each observed in single VSCC samples (Figure 1). None of the 27 samples displayed gain at 5p, the most frequent genetic alteration previously reported in a series of CxSCC analysed using an identical protocol.

### Comparison with previous studies on vulval neoplasia

This study is the first to use a high-resolution array-based technique to analyse the genetic alterations associated with hrHPV-positive VIN and VSCC. Indeed, few studies have previously examined the genetic changes associated with high-grade VIN using lower resolution techniques. One investigation (Rosenthal *et al*, 2001) reported that, of 6 loci (3p, 4q, 5p, 9p, 11p and 17p) examined for LOH using microsatellite marker analysis, 3p was the most frequently affected with LOH in 8 of 28 (29%) informative HPV-positive VIN3 samples, comparable to this study. Elsewhere, metaphase CGH was used to analyse the genetic events in nine HPV-positive VIN3 samples (Bryndorf *et al*, 2004). In line with this study, gains of whole chromosomes 1 and 20 were the most frequent aberrations observed in six (67%) and five (56%) of nine VIN3 samples, respectively. In contrast, gains at chromosome 19 were not detected; however, metaphase CGH has been shown to be unreliable for analysis of chromosome 19 aberrations (Kallioniemi *et al*, 1994).

The genetic alterations associated with hrHPV-positive VSCC have been analysed previously in several studies using various lower resolution techniques (summarised in Supplementary Table 2). Differences exist between these earlier investigations both in the nature of the samples (formalin-fixed paraffin embedded *vs* fresh frozen) and the methodologies used (microsatellite marker analysis that solely detects LOH *vs* metaphase CGH that has greater sensitivity for gain events). Nevertheless, all seven previous studies identified loss at 3p as an important event, occurring in 14% (1 out of 7) to 67% (4 out of 7) of samples, and recurrent gain of 8q was observed in all five CGH studies in 20% (2 out of 10) to 67% (4 out of 6) samples. Direct comparisons with our data are limited by methodological differences; however, both of these findings were consistent with our data. Some dissimilarities were apparent, in particular the lower frequency of gains reported elsewhere at chromosomes 1, 19 and 20. One explanation for these discrepancies may be the comparatively low overall level of the gains; in our samples the copy number ratios of these gains were often considerably lower than 1.5, suggesting that the frequency of these events may have been underestimated elsewhere had a higher cutoff value been used. Alternatively, because the low copy number ratio implied that the gains were present in only a subset of tumour cells and the samples examined here were early stage (Stage IA/B) cancers, it is possible that this subpopulation might decrease during VSCC progression. This would be consistent with data from one previous study showing a high frequency of chromosome 1 and 20 gains in VIN3 but not VSCC samples (Bryndorf *et al*, 2004). The fact that another investigation (Huang *et al*, 2005) observed gains at chromosomes 1 and 20 in only 17% of metastatic tumours provides further support for this theory. Although the remaining studies provided no information on tumour stage, the authors reported frequent recurrent gains at 3q and 5p, which have been shown to correlate with pathological staging of SCC (Oga *et al*, 2001; Yen *et al*, 2001; Noguchi *et al*, 2003; Klussmann *et al*, 2009); hence, it seems likely that many of these samples were also late stage tumours. Finally, it should be noted that two of the CGH studies included in the comparison (Jee *et al*, 2001; Micci *et al*, 2003) did not investigate the HPV status of the VSCC analysed; hence, it remains unclear whether their results are relevant to HPV-associated vulval neoplasia.

### Vulval and CxSCC may be associated with different genetic alterations

Our data suggest that vulval and cervical tumours may exhibit different patterns of chromosomal aberrations. Specifically, reanalysis of previously published CxSCC data revealed frequent recurrent gain at 5p. Other studies have reported a similar finding in early stage cervical tumours. For example, Wilting *et al* (2006) identified recurrent 5p gain in 33% (3 out of 9) non-metastatic CxSCC and Scotto *et al* (2008b) reported that 26% (5 out of 19) of high-grade CIN samples showed gain at 5p. In contrast, none of the series of 27 high-grade VIN and VSCC in this study displayed this aberration. Statistical analysis revealed a significant difference between CxSCC and VSCC samples with respect to 5p gain, although any definite conclusions must be limited by the small number of VSCC examined. After controlling for multiple testing, the other differences observed between the two tumour groups were not significant. However, it would be of interest to investigate some of these differences further in a larger series of vulval samples. For example, 3q gain was detected in 17% (1 out of 6) VSCC compared with 70% (37 out of 53) of the CxSCC series analysed concomitantly (including 60%, 6 out of 10 of the Stage I tumours) and 100% (9 out of 9) of the Stage I and II cervical tumours investigated by Wilting *et al* (2006). Furthermore, frequent recurrent gain of 3q has been reported in cervical neoplasia before the acquisition of the invasive phenotype: Kirchhoff *et al* (1999) and Umayahara *et al* (2002) identified 3q gain in 35% (6 out of 17) and 61% (11 out of 18), respectively, of CIN3 samples compared with 14% (3 out of 21) high-grade VIN in this study. These combined data suggest that chromosomal aberrations characteristic of advanced SCC at several sites, including hrHPV-associated cancers such as tonsillar SCC (Oga *et al*, 2001; Yen *et al*, 2001; Dahlgren *et al*, 2003; Noguchi *et al*, 2003; Klussmann *et al*, 2009), commonly appear at an early stage of cervical transformation.

It is well established that the incidence of cervical carcinoma is 5- to 10-fold greater than that of other lower genital tract cancers and peaks at least 10 years earlier (Watson *et al*, 2008). HPV infections are commonly detected throughout the region (Hording *et al*, 1996), implying that cervical epithelium may be more susceptible to transformation than other anogenital epithelia. The cervix bears a unique anatomical feature, the transformation zone, where squamous metaplasia occurs as a normal physiological phenomenon. It is this area that is particularly susceptible to HPV infection and where both infection and CIN begin. The reasons for this remain unknown. This may be because the cellular microenvironment of replicating non-maturing reserve cells or unique cell-surface receptors on this cell population favour HPV infection (Crum, 2000). The prevalence of HPV-18 infection is higher in cervical than in other anogenital SCC (De Vuyst *et al*, 2009), and it has been speculated that HPV-18 infection may be more aggressive clinically (Bulk *et al*, 2007). However, differences in type-specific prevalence are not sufficiently great to account for the disparity in cancer incidence. Another possible explanation is that the susceptibility to hrHPV-mediated transformation of the various anogenital epithelia may in part reflect their immune or hormonal microenvironment. It has recently been found that the clinical outcome of preinvasive cervical lesions can be accurately predicted by the physical status of the hrHPV genome (integrated *vs* episomal) in conjunction with viral load (Wanram *et al*, 2009). The transition from low-grade dysplastic lesion harbouring episomal genomes to late-stage invasive carcinoma containing only integrated copies has been shown to occur by an intermediate step in which episomal and integrated genomes co-exist (Pett *et al*, 2004). Cells harbouring both genome types were characterised by copy number imbalances that predominantly comprised whole chromosome gains or losses, similar to the genetic events observed in vulval neoplasia in this study. In contrast, cells containing HPV integrant alone displayed frequent structural as well as numerical chromosomal instability, with recurrent aberrations including gain at 3q and 5p, mirroring those identified in the cervical tumours analysed here as well as in high-grade CIN (Scotto *et al*, 2008b). During the intermediate stage, integrants are subject to transcriptional repression by the episomal genomes (Dall *et al*, 2008). Selective advantage has been found to be insufficient for the progression of the integrant-bearing cell (Pett *et al*, 2006); rather there appears to be a requirement for episomal clearance, which can be hastened by treatment with various immunomodulators including type I interferon (Herdman *et al*, 2006). The physical status of the hrHPV genome in vulval neoplasia remains unclear. Data suggest that HPV integrants are present in a lower proportion of high-grade VIN than CIN (Hudelist *et al*, 2004; Hillemanns and Wang, 2006). The presence in VSCC of both integrated and episomal forms has been shown (Ueda *et al*, 2004), although analysis was restricted to a single sample. A larger study identified HPV integrants in the majority of VSCC examined (van de Nieuwenhof *et al*, 2009); however, the possibility of co-existent viral episomes was not excluded. Further work is clearly required to examine this hypothesis.

### HPV-associated VSCC display HPV-specific aberrations

Previous studies have reported significant genetic differences between hrHPV-associated and hrHPV-independent SCC at a range of anatomical sites, including the oral cavity, oropharynx and cervix (Dahlgren *et al*, 2003; Braakhuis *et al*, 2004; Smeets *et al*, 2006; Klussmann *et al*, 2009; Wilting *et al*, 2009). The alterations identified as specific for hrHPV-independent tumours, namely recurrent losses at 5q and 9p and gains at 7q and 11q, were not observed in our VSCC series. Conversely, recurrent gain at chromosome 20, observed in 67% (4 out of 6) VSCC, was found to be a specific marker of HPV-associated cancer. All five earlier studies also reported that hrHPV-positive SCC were characterised by a lower level of chromosomal aberrations overall. Our laboratory has performed preliminary analysis (data not shown) on three HPV-independent VSCC, categorised thus because tumours developed in a background of lichen sclerosus and/or differentiated VIN and tested negative for hrHPV DNA. These data suggest that the significant genetic differences between hrHPV-positive and hrHPV-negative tumours observed at other sites may be mirrored in the vulva. The most marked difference was the finding of 9p loss in all three HPV-independent tumours. In addition, allelic imbalance was observed at a mean number of 10 chromosomes, compared with 6.5 in the HPV-associated VSCC. In contrast to these data, an earlier study from our laboratory (Purdie *et al*, 2009) examining the genetics of cutaneous SCC found no significant differences between HPV-positive and HPV-negative tumours. However, it must be noted that skin cancers are overwhelmingly associated with HPV from the *β* rather than *α* genus. The most common recurrent chromosomal aberration identified in cutaneous SCC was LOH at 9p, with similar frequencies in HPV-positive and HPV-negative SCC (32 out of 43, 74% and 13 out of 17, 76% respectively), whereas gain at chromosome 20 was observed at much lower frequency in 16% (7 out of 43) and 18% (3 out of 17) of HPV-positive and HPV-negative tumours, respectively. These combined data suggest that HPV from the *β* and *α* genera are associated with distinct patterns of chromosomal aberrations.

### Possible candidate genes targeted by genetic alterations

Despite considerable methodological differences, combined data from the current and previous investigations suggest a characteristic pattern of genetic changes within VSCC. In particular, deletion at 3p was reported as a frequent recurrent event in all studies. This aberration is likely to target fragile histidine triad (*FHIT*), a recognised tumour suppressor gene known to be inactivated in other SCC (Yoshino *et al*, 2000; Lee *et al*, 2006; Purdie *et al*, 2009). Indeed, one of the VSCC samples exhibited a microdeletion restricted to the *FHIT* locus at 3p14.2. *FHIT* is located at the fragile site *FRA3B*, which has been identified as an integration hotspot for HPV (Huang *et al*, 2005), although *FHIT* also appears to be targeted during HPV-independent carcinogenesis. Gain at 8q, resulting in amplification of the oncogene *c-Myc* (Issing *et al*, 1993), was also consistently identified, as was gain at chromosome 20, a specific marker of HPV-associated cancer that has been reported to result in elevated mRNA levels of the *de novo* DNA methyltransferase *DNMT3B* at 20q (Wilting *et al*, 2006). *In vitro* studies (Savelieva *et al*, 1997; Klingelhutz *et al*, 2005) have shown that gain at 20q is an early event in hrHPV-mediated immortalisation of epithelial cells, suggesting that increased DNMT3B expression may contribute to the sequential tumour suppressor gene promoter hypermethylation that has been identified during HPV-induced transformation (Henken *et al*, 2007). Overall, the most frequent event across our series was gain at 1p, shown elsewhere to correlate with increased expression of the *JUN* protooncogene (Taniguchi *et al*, 2007). Gain at 3q, a marker of late stage HPV-associated cancer, was less frequently observed in the VSCC series. This aberration has been shown to result in the amplification of *hTERC*, the RNA component of human telomerase (Heselmeyer-Haddad *et al*, 2003), thereby complementing the activity of the hrHPV E6 oncoprotein that transcriptionally activates *hTERT*, the catalytic subunit of telomerase (Veldman *et al*, 2001). Uniparental disomy at 6p was the only recurrent copy-neutral LOH event identified in the series. Although this aberration was restricted to VIN samples, VSCC numbers were too small to determine whether this represented a genuine difference between preinvasive and invasive lesions. In cervical neoplasia, LOH at 6p is frequent in both high-grade CIN and CxSCC (Chatterjee *et al*, 2001; Kloth *et al*, 2007). Evidence suggests that HLA class I genes are the target of this aberration (Koopman *et al*, 2000), thereby permitting tumours to escape immune surveillance.

In summary, we have used high-resolution SNP microarray analysis to investigate genome-wide allelic imbalance in hrHPV-associated vulval neoplasia. Across the spectrum, gains of chromosomal material were more frequent than losses. Despite shared HPV status and regional proximity, our data suggest that vulval and cervical tumours may differ in their pattern of associated genetic alterations. A better understanding of any differences is likely to provide insight into mechanisms of HPV-associated epithelial carcinogenesis and may prove important in future studies of diagnostic, predictive and therapeutic biomarkers relevant to VSCC treatment and prevention.

## Figures and Tables

**Table 1:** Recurrent aberrations in VIN and VSCC

		**Frequency** a	
**Chromosome**	**Copy number change**	**VIN (*n*=21)**	**VSCC (*n*=6)**
1p	Gain	13 (62%)	6 (100%)
1q	Gain	10 (48%)	3 (50%)
2q	Deletion	4 (19%)	2 (33%)
3p	Deletion	4 (19%)	2 (33%)
6p	UPD	5 (24%)	0
8p	Gain	4 (19%)	2 (33%)
8q	Gain	5 (24%)	3 (50%)
9p	Gain	5 (24%)	2 (33%)
9q	Gain	8 (38%)	3 (50%)
10p	Deletion	6 (29%)	1 (17%)
10q	Deletion	5 (24%)	1 (17%)
14q	Deletion	1 (5%)	2 (33%)
16p	Gain	7 (33%)	0
16q	Gain	6 (29%)	0
19	Gain	13 (62%)	3 (50%)
20p	Gain	14 (66%)	4 (67%)
20q	Gain	14 (66%)	4 (67%)
22q	Gain	9 (43%)	2 (33%)
Xp	Deletion	1 (5%)	2 (33%)
Xp	Gain	8 (38%)	1 (17%)
Xq	Gain	7 (33%)	1 (17%)

Abbreviations: UPD=uniparental disomy or copy-neutral loss of heterozygosity; VIN=vulval intraepithelial neoplasia; VSCC=vulval squamous cell carcinoma.

a Events are only listed if they occurred in 20% or more of one sample group.

**Table 2:** Comparison of aberrations in VSCC and CxSCC

**Event**	**VSCC**	**All CxSCC** a	**Stage I**^b^ **CxSCC**
Gain at 1p	6/6 (100%)	24/53 (45%)	4/10 (40%)
Gain at 1q	3/6 (50%)	29/53 (55%)	5/10 (50%)
Loss at 2q	2/6 (33%)	22/53 (43%)	3/10 (30%)
Loss at 3p	2/6 (33%)	34/53 (64%)	4/10 (40%)
Gain at 3q	1/6 (17%)	37/53 (70%)	6/10 (60%)
Loss at 4p	1/6 (17%)	26/53 (49%)	6/10 (60%)
Loss at 4q	0/6	24/53 (45%)	7/10 (70%)
Gain at 5p	0/6	31/53 (58%)	5/10 (50%)
Loss at 5q	1/6 (17%)	17/53 (32%)	2/10 (20%)
Gain at 6p	0/6	13/53 (25%)	3/10 (30%)
Gain at 7q	3/6 (50%)	10/53 (19%)	2/10 (20%)
Gain at 8p	2/6 (33%)	16/53 (30%)	2/10 (20%)
Gain at 8q	3/6 (50%)	23/53 (43%)	2/10 (20%)
Loss at 9p	0/6	10/53 (19%)	2/10 (20%)
Gain at 9q	3/6 (50%)	14/53 (26%)	1/10 (10%)
Loss at 11q	1/6 (17%)	24/53 (45%)	3/10 (30%)
Loss at 13q	1/6 (17%)	23/53 (43%)	2/10 (20%)
Loss at 14q	2/6 (33%)	2/53 (4%)	0/10 (0%)
Gain at 15q	0/6	16/53 (30%)	1/10 (10%)
Gain at 19q	3/6 (50%)	27/53 (50%)	6/10 (60%)
Gain at 20q	4/6 (67%)	22/53 (42%)	3/10 (30%)
Gain at 22q	2/6 (33%)	13/53 (25%)	2/10 (20%)

a Reanalysed data from a previous study on cervical carcinomas (Scotto *et al*, 2008b).

b According to FIGO criteria, Stage I CxSCC is carcinoma restricted to the cervix.
