# Supplementary material for: High-resolution genomic profiling of human papillomavirus-associated vulval neoplasia
Source: Br J Cancer. 2010 Mar 16;102(6):1044–51. doi: 10.1038/sj.bjc.6605589 (PMC2844038; doi:10.1038/sj.bjc.6605589)
Supplement: Supplementary Table 2 [file 6605589x2.doc]

| **SUPPLEMENTARY TABLE 2. Summary of genetic changes associated with vulval SCC** | | | | | | | | |
| --- | --- | --- | --- | --- | --- | --- | --- | --- |
| Event | Pinto et al 1999 | Jee et al 2001 | Rosenthal et al 2001 | Allen et al 2002 | Micci et al 2003 | Bryndorf et al 2004 | Huang et al 2005 | Current study |
| -1p | 2/6; 33% |  |  |  |  |  |  |  |
| +1p |  |  |  |  | 3/7; 43% |  | 1/6; 17% | 6/6; 100% |
| -1q | 2/8; 25% |  |  |  |  | 1/6; 17% |  |  |
| +1q |  |  |  |  | 1/7; 14% |  | 1/6; 17% | 3/6; 50% |
| -2p | 0/4 |  |  | 1/10; 10% |  | 1/6; 17% |  | 1/6; 17% |
| +2p |  |  |  |  | 1/7; 14% |  |  | 1/6; 17% |
| -2q | 4/6; 67% |  |  | 1/10; 10% |  | 1/6; 17% |  | 2/6; 33% |
| -3p | 3/6; 50% | 4/10; 40% | 7/32; 22% | 4/10; 40% | 1/7; 14% | 4/6; 67% | 3/6; 50% | 2/6; 33% |
| -3q |  |  |  | 1/10; 10% |  | 1/6; 17% |  |  |
| +3q |  | 4/10; 40% |  | 5/10; 50% | 3/7; 43% | 4/6; 67% | 2/6; 33% | 1/6; 17% |
| -4p | 2/6; 33% | 5/10; 50% |  | 1/10; 10% | 2/7; 29% | 1/6; 17% | 1/6; 17% | 1/6; 17% |
| -4q |  |  | 5/33; 15% |  |  |  | 1/6; 17% |  |
| -5p |  |  | 5/31; 16% |  |  | 3/6; 50% |  | 1/6; 17% |
| +5p |  |  |  | 2/10; 20% | 2/7; 29% |  | 3/6; 50% |  |
| -5q | 1/8; 13% | 2/10; 20% |  | 1/10; 10% |  | 1/6; 17% |  | 1/6; 17% |
| +6p |  |  |  |  | 1/7; 14% | 3/6; 50% |  |  |
| -6q |  | 1/10; 10% |  | 1/10; 10% |  |  |  |  |
| +6q |  |  |  |  |  | 1/6; 17% |  |  |
| -7p |  |  |  |  | 1/7; 14% |  |  |  |
| +7p |  |  |  |  |  | 1/6; 17% | 2/6; 33% | 1/6; 17% |
| -7q |  |  |  | 1/10; 10% | 1/7; 14% | 1/6; 17% | 1/6; 17% |  |
| +7q |  | 1/10 10% |  |  |  |  |  | 3/6; 50% |
| -8p | 2/6; 33% |  |  | 1/10; 10% | 1/7; 14% | 2/6; 33% |  | 1/6; 17% |
| +8p |  | 1/10; 10% |  |  |  |  |  | 2/6; 33% |
| -8q | 3/6; 50% |  |  |  |  |  |  |  |
| +8q |  | 3/10; 30% |  | 2/10; 20% | 3/7; 43% | 2/6; 33% | 4/6; 67% | 3/6; 50% |
| -9p |  |  | 10/28; 36% |  |  |  |  |  |
| +9p |  | 1/10; 10% |  |  | 1/7; 14% | 1/6; 17% |  | 2/6; 33% |
| +9q |  |  |  |  | 2/7; 29% | 2/6; 33% | 1/6; 17% | 3/6; 50% |
| -10p | 1/6: 17% |  |  | 1/10; 10% | 2/7; 29% | 2/6; 33% |  | 1/6; 17% |
| -10q | 1/4; 25% |  |  | 1/10; 10% | 1/7; 14% | 1/6; 17% | 1/6; 17% | 1/6; 17% |
| -11p | 3/7; 43% |  | 10/41; 24% | 1/10; 10% | 1/7; 14% | 2/6; 33% | 1/6; 17% | 1/6; 17% |
| +11p |  |  |  |  | 1/7; 14% |  |  |  |
| -11q | 2/8; 25% | 1/10; 10% |  | 4/10; 40% | 3/7; 43% | 3/6; 50% | 2/6; 33% | 1/6; 17% |
| +11q |  |  |  |  |  | 1/6; 17% |  | 1/6; 17% |
| -12p | 0/7 |  |  |  |  | 1/6; 17% |  |  |
| +12p |  |  |  |  |  | 1/6; 17% |  |  |
| -12q |  |  |  |  |  | 1/6; 17% |  |  |
| +12q |  |  |  |  |  | 1/6; 17% |  |  |
| -13q |  | 1/10; 10% |  | 1/10; 10% | 1/7; 14% | 1/6; 17% |  | 1/6; 17% |
| -14q | 2/6; 33% |  |  |  | 1/7; 14% |  | 1/6; 17% | 2/6; 33% |
| +14q |  | 1/10; 10% |  |  | 1/7; 14% | 1/6; 17% |  |  |
| -15q | 3/7; 43% |  |  |  | 1/7; 14% | 2/6; 33% |  |  |
| +16p |  |  |  |  | 1/7; 14% | 1/6; 17% |  |  |
| -16q |  |  |  | 1/10; 10% |  |  | 1/6; 17% |  |
| +16q |  |  |  |  | 1/7; 14% | 1/6; 17% |  |  |
| -17p | 3/7; 43% |  | 11/36; 31% |  | 1/7; 14% | 1/6; 17% | 1/6; 17% |  |
| +17p |  | 1/10; 10% |  |  |  |  |  |  |
| -17q |  |  |  |  |  | 1/6; 17% | 1/6; 17% |  |
| -18p | 1/6; 16% |  |  |  |  | 1/6; 17% |  |  |
| +18p |  |  |  |  | 1/7; 14% |  |  |  |
| -18q | 1/8; 13% |  |  | 2/10; 20% | 1/7; 14% | 1/6; 17% |  |  |
| +19p |  |  |  |  |  |  |  | 3/6; 50% |
| +19q |  |  |  |  | 2/7; 29% |  | 1/6; 17% | 3/6; 50% |
| +20p |  |  |  |  |  | 1/6; 17% | 1/6; 17% | 4/6; 67% |
| -20q |  |  |  |  |  | 16; 17% |  |  |
| +20q |  | 1/10; 10% |  | 1/10; 10% | 1/7; 14% | 1/6; 17% | 1/6; 17% | 4/6; 67% |
| -21q | 4/6; 67% |  |  |  |  |  |  |  |
| +21q |  |  |  |  |  | 1/6; 17% |  | 1/6; 17% |
| -22q | 1/5; 20% |  |  |  |  |  | 1/6; 17% |  |
| +22q |  |  |  |  | 1/7; 14% |  |  | 2/6; 33% |
| Comment | MM^a^ at 21 loci in 8 HPV-positive FFPE VSCC^b^; loci not always informative | mCGH^c^ on10 FFPE VSCC (9 well differentiated) | MM on 6 loci in 47 HPV-positive FFPE VSCC; loci not always informative | mCGH on10 HPV-positive FFPE VSCC | mCGH on 7 fresh frozen VSCC, all moderately/ poorly differentiated | mCGH on 6 HPV-positive FFPE VSCC | mCGH on 6 HPV-positive fresh-frozen VSCC (all late stage III/IV) | SNP array analysis of 6 HPV-positive fresh frozen VSCC all early stage (Ia/Ib) |

^a^MM, microsatellite marker analysis, blank rows indicate event not examined

^b^Unless specified no information was provided on tumour stage, differentiation status or HPV typing

^c^mCGH, metaphase comparative genomic hybridisation, blank rows indicate event not observed
